# MBAT: A scalable informatics system for unifying digital atlasing workflows

**DOI:** 10.1186/1471-2105-11-608

**Published:** 2010-12-22

**Authors:** Daren Lee, Seth Ruffins, Queenie Ng, Nikhil Sane, Steve Anderson, Arthur Toga

**Affiliations:** 1 UCLA Laboratory of NeuroImaging, David Geffin School of Medicine, Los Angeles, CA USA

## Abstract

**Background:**

Digital atlases provide a common semantic and spatial coordinate system that can be leveraged to compare, contrast, and correlate data from disparate sources. As the quality and amount of biological data continues to advance and grow, searching, referencing, and comparing this data with a researcher's own data is essential. However, the integration process is cumbersome and time-consuming due to misaligned data, implicitly defined associations, and incompatible data sources. This work addressing these challenges by providing a unified and adaptable environment to accelerate the workflow to gather, align, and analyze the data.

**Results:**

The MouseBIRN Atlasing Toolkit (MBAT) project was developed as a cross-platform, free open-source application that unifies and accelerates the digital atlas workflow. A tiered, plug-in architecture was designed for the neuroinformatics and genomics goals of the project to provide a modular and extensible design. MBAT provides the ability to use a single query to search and retrieve data from multiple data sources, align image data using the user's preferred registration method, composite data from multiple sources in a common space, and link relevant informatics information to the current view of the data or atlas. The workspaces leverage tool plug-ins to extend and allow future extensions of the basic workspace functionality. A wide variety of tool plug-ins were developed that integrate pre-existing as well as newly created technology into each workspace. Novel atlasing features were also developed, such as supporting multiple label sets, dynamic selection and grouping of labels, and synchronized, context-driven display of ontological data.

**Conclusions:**

MBAT empowers researchers to discover correlations among disparate data by providing a unified environment for bringing together distributed reference resources, a user's image data, and biological atlases into the same spatial or semantic context. Through its extensible tiered plug-in architecture, MBAT allows researchers to customize all platform components to quickly achieve personalized workflows.

## Background

Digital atlases provide semantic and spatial information that can be used to link together rich collections of data from disparate sources [1]. The most common types are anatomic atlases that spatially delineate and semantically label the structures of a volumetrically imaged subject. These anatomic atlases can be used as templates for identifying regions of interest in non-delineated data sets, such as localizing gene expression in the brain as visualized by in situ hybridization [2,3]. Atlases can also be used to unify implicitly associated data. For example, one data source may use an anatomical name for a location while another uses image coordinates. By aligning both data to a common atlas, correlations can be made. As the quality and amount of biological data continues to advance and grow, having the ability to search, reference, and compare this data with a researcher's own data is essential.

**Figure 1:**
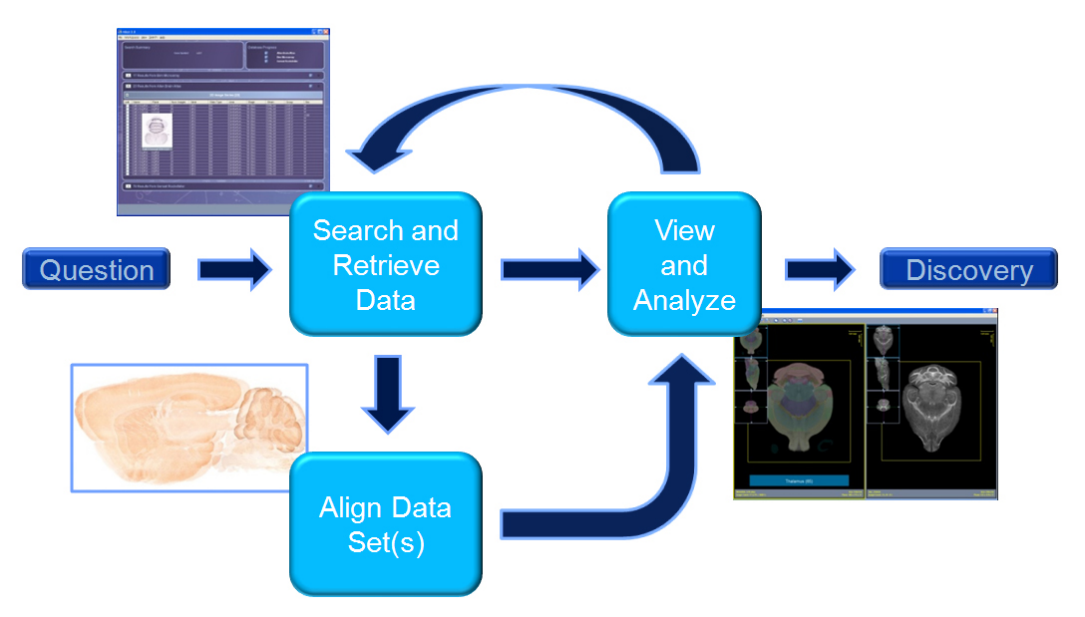
**Digital Atlas Analysis Workflow**. In the first stage, a researcher searches and retrieves data to help answer the proposed question. The data can be of different types and from multiple sources. If necessary, the data is aligned to a common space, either to a template, such as an atlas, or to each other. In the next stage, the researcher compares and contrasts the data. A researcher can iteratively repeat this process as needed.

Central to integrating and correlating the continually growing volumes of distributed and online data is the atlas analysis workflow. As shown in Figure 1, the atlas analysis workflow consists of three major steps: (1) searching and retrieving source data, (2) aligning the source data to the atlas, and (3) comparing and correlating the data in context of the atlas space. This process can be repeated multiple times as interesting or unexpected correlations spark new ideas and findings.

Several challenges exist with the digital atlas workflow that make conducting the analysis a cumbersome and time-consuming process. First, each data source typically has a different search interface and result format which makes reconciling data difficult. Users must use different interfaces, such as visiting separate web sites, and utilize different software tools to view and analyze the data. Second, spatially aligning or registering image data to an atlas or to each other is a complex problem with many solutions. Factors that influence what type of registration algorithms to use include image modality of the data, dimensionality of the source and template data (2D-2D, 3D-3D, or 2D-3D), speed or time requirements, and accuracy, such as whole brain or structure accuracy [4]. Making assumptions about any of these factors limits the generality and usefulness of the overall application. Third, although visually comparing and contrasting the data is a natural and intuitive method of analysis, few computer applications can integrate image data, numerical data, annotations, ontological relationships, and atlas data in a meaningful manner.

To address these issues, this work presents the MouseBIRN Atlasing Toolkit (MBAT) - a cross-platform, free open-source software tool designed to accelerate the timeline for integrating and correlating biological data. MBAT empowers researchers to discover correlations among disconnected and disparate data by providing a unified environment for bringing together distributed reference resources, a user's image data, and biological atlases into the same spatial or semantic context. In a single application, MBAT provides the ability to use a single query to search and retrieve data from multiple data sources, align image data using the user's preferred registration method, composite data from multiple sources in a common space, and link relevant information to the current view of the data or atlas. Through its extensible tiered plug-in architecture, MBAT allows researchers to customize all application components to quickly achieve personalized workflows.

### Related Work

As shown in Table 1, related cross-platform, free open-source software include an atlas navigator, JAtlasViewer [5], and two medical imaging analysis tools with plug-in architectures, Medical Imaging Processing Analysis and Visualization (MIPAV) [6] and Slicer [7].

**Table 1:** Comparison of related software.

Name	Search	Registration	Visualization	Comparison	Atlas	Extendible
JAtlas Viewer	None	None	Single 3D image and surface view; oblique image view; segmented surfaces	None	View labels as text and segmented color; label selection; hierarchy view;	No
MIPAV	None	B-spline; Landmark; Cost optimization	3D and orthogonal views; oblique view; volume rendering	Juxtapose Image sets (no over- lay); Synchronized view	None	PlugInAlgorithm; PlugInFile; PlugInView
Slicer	QueryAtlas Module (publication search only)	Linear Module; Register Images Module	3D and orthogonal views; oblique view; volume rendering	Overlay image sets; view image sets together in 3D space	Overlay labels as image (only label ID available)	Modules

Comparison of cross-platform, free open-source software for search, registration, image comparison, atlas viewing, and extensibility features.

JAtlasViewer is a lightweight Java application that provides arbitrary section views of 3D image data, 3D surfaces of pre-segmented and labeled anatomy, and anatomical browsing. Only a single data set can be loaded at a time so it lacks the ability to perform comparisons among data sets. It also lacks any search and registration features.

MIPAV and Slicer are more mature, larger efforts to build a suite of tools for medical image analysis. Both provide powerful visualization, registration, and analysis features. However, only Slicer provides some limited labeling and search functionality. In the Volume module, the label volumes can be overlaid on image volumes but only the label id can be interrogated; the corresponding label name and color are not supported. In the QueryAtlas module, label names and colors are displayed on 3D surfaces or sectional views, with links to several ontologies, but the query engine only supports literature sources. While MIPAV supports plug-ins, the types of plug-ins are limited to image processing algorithms, file format readers, and rendering methods. Slicer's plug-ins, called modules, offer greater flexibility as the modules can be implemented as shared libraries or leverage existing legacy executables. To simplify the development, Slicer also provides methods to auto-generate GUIs for the plug-ins. However, Slicer's architecture is based on manipulating objects in a global 3D scene so adding non-graphical objects, such as search objects, to Slicer is cumbersome.

What sets our work apart from the others are three key features. First, our framework has the ability to integrate multiple data types from multiple, disparate sources. Gathering and searching for data must be performed external to the other applications. Second, our framework adopts the plug-in architecture from top to bottom and is designed for interoperability. Parts of other applications can be integrated into our application and vice versa, parts of our application can be integrated into other applications. Third, novel digital atlasing features are developed, such as supporting multiple label sets, dynamic selection and grouping of labels, and synchronized display of ontological data.

## Implementation

The core philosophy behind our framework design is to provide large-scale extensibility. Since the details of each analysis step varies from field to field and by investigator, our goal is to ensure that each component and subcomponent in the atlas analysis workflow can be extended or customized without major rewriting of the core application components. To achieve this goal, a modular and tiered plug-in architecture is adopted and employed. In this paper, we present specific plug-in examples that illustrate the flexibility and scalability of our design. The current MBAT plug-ins focus on integrating the large and rich collections of mouse genomic and neuro-anatomical data that are being produced to study and understand various neurological disorders [8-11]. For cross-platform development, the framework is developed using the Java programming language.

### Tiered Plug-in Core Architecture

**Figure 2:**
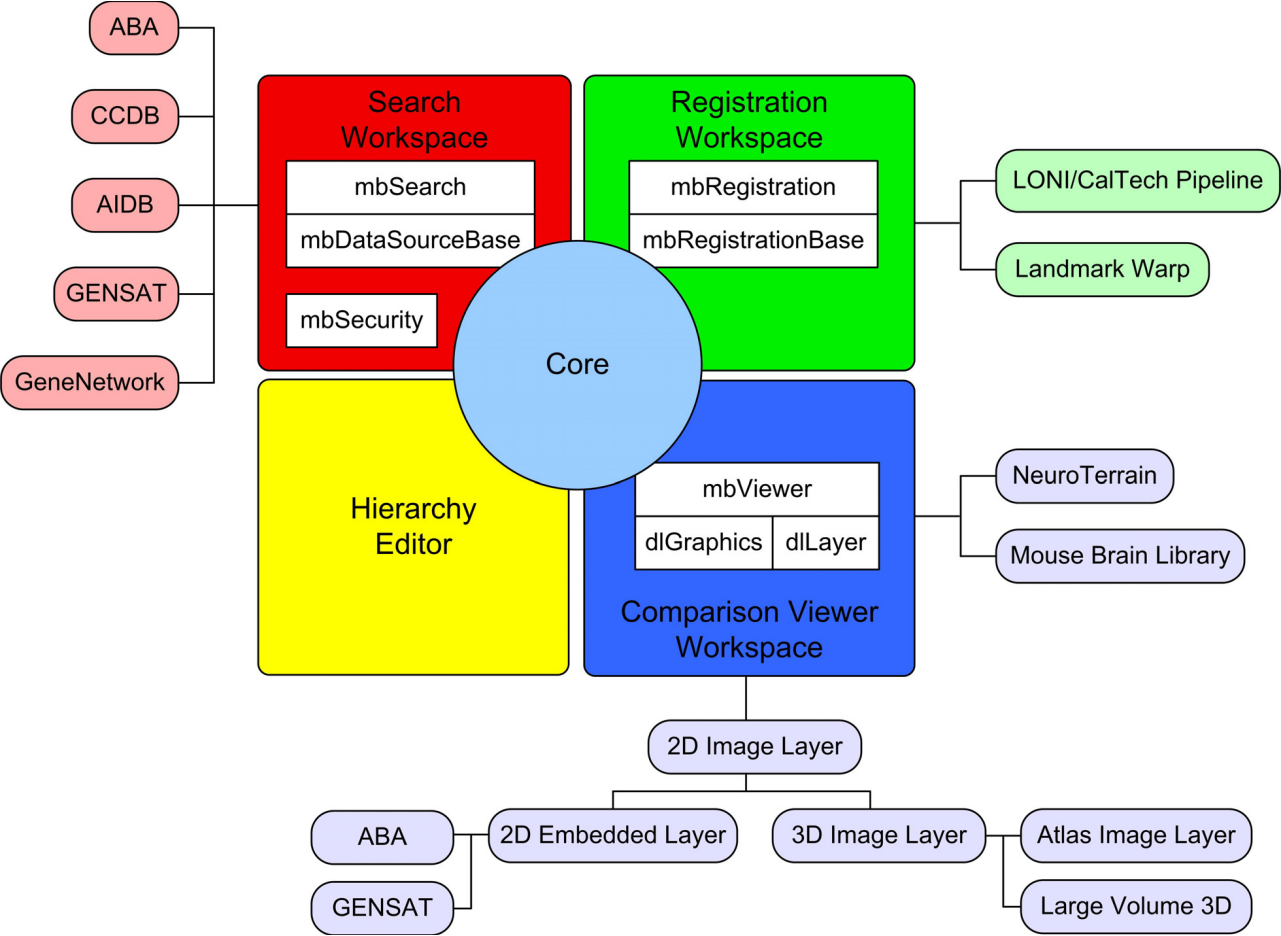
**MBAT Workspace Architecture**. For the neuroinformatics and genomics tool, MBAT, the core framework is extended to include four workspaces. The Search, Registration, and Comparison Viewer Workspaces use second-tier, tool plug-ins to customize the functionality of the workspace. The Hierarchy Editor Workspace is a stand-alone workspace with no plug-ins.

The design of the core architecture is built around customizable workspace plug-ins. Each workspace typically is used for a particular task, such as registering or viewing data, but in general, can be used for any combination of tasks. The functionality and design of these workspaces are entirely driven by the end user's goals. To achieve this flexibility, a tiered, plug-in architecture is adopted. The workspace or first-tier plug-ins can provide the entire functionality or can be further customized using second-tier or tool plug-ins. The benefits of a plug-in architecture are (1) subcomponents can be added or extended without making major changes to the main system and (2) it enforces a high degree of modularity in the design, leading to a highly scalable architecture. As evidenced by the use of ImageJ [12], an image processing tool that heavily uses plug-ins, this modularity allows users to adapt and tailor the core functionality to meet their own specific needs. This has led to a large scientific community of ImageJ users who share, exchange, and extend each other's plug-ins [13]. The core framework was tailored into a neuroinformatics application, MBAT, that is built around the workspaces of search, registration, and comparison viewing. The overall architecture for the MBAT workspaces, shown in Figure 2, is described below.

### Search Workspace

The primary goal of the Search Workspace is to provide a federated search where a single query is used to search across multiple data sources, making the underlying heterogeneous data services transparent to the user. The major challenges addressed by this work include integrating all the non-standardized services that each data source provides and displaying the results in an intuitive manner that allows for easy browsing and selection for further analysis. For the federated search, the query process is divided into three tasks: (1) specifying the search parameters, (2) executing the query on each data source, and (3) displaying the query results for browsing. The core search engine manages the first and last tasks; the task of executing the query is delegated to data source plug-ins. This design gives the plug-ins full control over the query logic since executing the actual query depends on the underlying database system and will vary from data source to data source. Moreover, this modular data source plug-in design provides great flexibility since it imposes no restrictions on the data sources or data access method. The underlying data source can use any database system, any query language, and it can be local or remote. In this way, our search engine can connect to existing data sources without any modifications to the source as well include new data sources as they become available. Each data source can use any data access method, such as through web services, direct connection to databases, file systems, or remote streaming servers. Through extension of rendering layer plug-ins, custom plug-ins can act as middleware to deliver the data from the source location into MBAT.

### Registration Workspace

The aim of the Registration Workspace is to allow users to align their data to a template such as an atlas, or to each other so meaningful comparison can then be made. However, because there are many registration algorithms for different purposes with different strengths and weaknesses, it is difficult to provide a universal solution that works well for all cases. To address this issue, the design of the Registration Workspace is built around registration algorithm plug-ins that can be selected by the user. In this way, researchers can choose or add the registration algorithm that best suits their needs.

Because the user interface and parameter requirements vary greatly from algorithm to algorithm, the core registration API only provides the interface for specifying the input source and template objects and saving of the aligned result object to the cart. Invoking the registration algorithm and providing the user interface to specify the user-defined parameters are delegated to the plug-in since each algorithm typically needs a different GUI and set of required arguments. Although the core registration API only provides input and output functionality, this design provides the greatest flexibility as it imposes no restrictions on the registration algorithm. For example, the algorithm can be written in Java or leverage the Java Native Interface (JNI) to execute native code. The registration algorithm can run locally or be executed on a remote machine. The system can launch the registration algorithm directly or launch a larger workflow that includes other steps, such as pre-processing the data. The registration plug-in can provide basic or complex user interfaces, customized either for the algorithm parameters or target users.

### Comparison Viewer Workspace

To allow users the ability to easily compare and contrast data, the Comparison Viewer Workspace is built around compositing layers of data. This compositing framework allows the user to easily juxtapose or overlay multiple datasets together for comparison. The major visualization challenge is integrating image data, numerical data, annotations, ontological relationships, and atlas data in a meaningful manner. Our approach is centered around a flexible, context-driven image viewer that synchronizes the relevant and associated informatics data to the current view of the image or atlas data. For example, to reference data against a digital atlas, the system will dynamically display the associated anatomical labels for the region of interest, highlight the current relationships in an ontology graph display, and list the annotations for the current data layer.

The tool plug-ins for the Comparison Viewer Workspace are divided into three categories - layer rendering, graph viewer, and analysis plug-ins. For the rendering and compositing, the functionality is divided between (1) a core graphics engine and (2) the layer rendering plug-ins. The core graphics engine handles adding, removing, compositing, linking, and transforming the layers, such as pan, rotate, and scaling. The rendering plug-ins handle loading of data, rendering the data content into the layer, and provide a GUI for controlling and adjusting the properties of the layer. By delegating the rendering of the data to the plug-in, customized display of any data type can be achieved in this modular design. This provides great flexibility in the manner in which image data is loaded into the layer. For example, out-of-core algorithms can be used to load data that is too large to fit into main memory or image servers can be leveraged to process data remotely and stream the image data over the network. The one restriction that is imposed is the rendering plug-in must use OpenGL to render the data. OpenGL and JOGL [14] were chosen since they are mature, cross-platform graphics libraries that leverage modern graphics hardware for increased performance.

The graph viewer plug-ins control how ontological data is displayed in conjunction with the layer rendering plug-ins. The display of the ontological data is divided into two tasks: (1) loading and parsing the ontology files and (2) rendering the graph visualization. The core graph viewer API provides methods to load and traverse the ontology hierarchy and supports the ILF MBAT hierarchy format and the Open Biomedical Ontologies (OBO) format [15]. The rendering of the ontology is delegated to the graph viewer plug-ins to allow customization of the graph visualization. Currently, the graph viewer plug-ins are used to display the ontology for an associated atlas. Each graph viewer plug-in is also responsible for receiving and sending synchronized graph events from and to the atlas layer in the Comparison Viewer. The graph events specify actions such as atlas structure selection and removal, structure hierarchy expansion and collapse, and sending the currently selected structure names to the Search Workspace.

The analysis plug-ins perform image processing tasks or statistical analysis on the data. To leverage the wealth of existing image analysis plug-ins for ImageJ, the Comparison Viewer framework has been designed to integrate with the ImageJ framework. As long as the active layer rendering plug-in stores or can convert the underlying image data to the basic ImageJ image structure, the ImageJ plug-ins can be applied. The MBAT Comparison Viewer Workspace loads and runs standard ImageJ plug-ins.

## Results

MBAT was developed as part of the Biomedical Informatics Research Network (BIRN, http://www.birncommunity.org) [16], a project focused on building an infrastructure for collaborative environments. The MouseBIRN testbed group consists of six partner institutions at Duke University, Drexel University, University of Tennessee, Memphis, California Institue of Technology, University of California, San Diego, and University of California, Los Angeles. UCLA LONI developed the core architecture and led the collaborative plug-in development among the other partners. In this section, a sample MBAT workflow is first presented to show the workspace interaction followed by a more detailed description of the plug-ins that were developed for each workspace.

### Use Case

**Figure 3:**
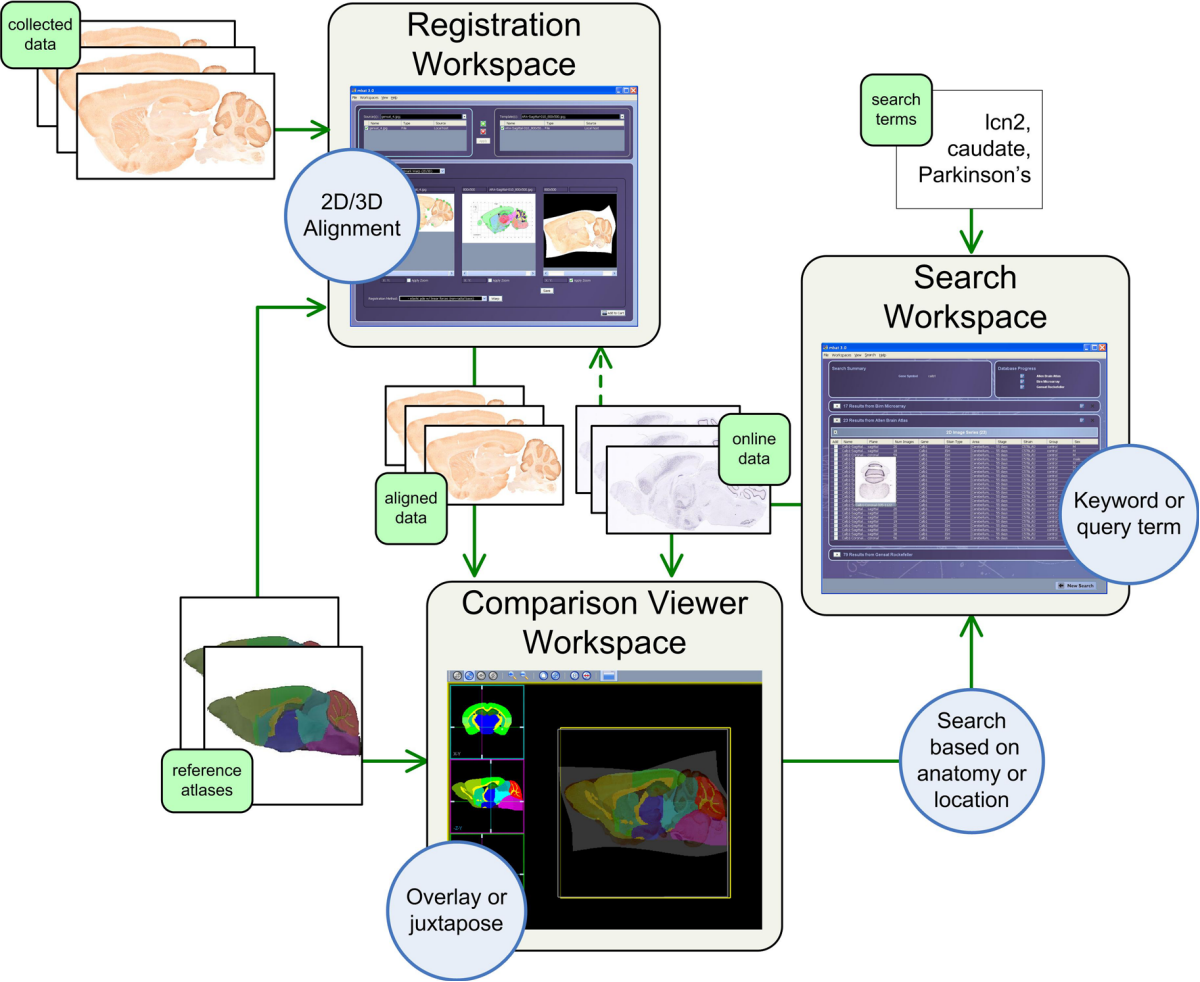
**MBAT workspace interaction**. Collected image data is first aligned with reference atlases and then overlaid or juxtaposed to identify the anatomic structures of regions of interest. Next, the researcher initiates a search for the anatomic structures or locations. In the Search Workspace, the online data sources and search criteria can be refined. The researcher can preview the results and select several for comparison. Returning back to the Comparison Viewer Workspace, the selected online images can be automatically pulled in and compared with the existing collected data and reference atlases. As new hypotheses are sparked by correlating data from multiple resources, this gathering, aligning, and correlating process can be repeated.

Consider a researcher who has collected a series of slices for an experiment for Parkinson's disease in relation to the Lipocalin-2 gene (lcn2) using a mouse model. During the analysis, the researcher finds an unusual formation in a part of the brain that he/she has difficultly identifying. To help identify the region of interest, the researcher would like to compare and contrast the collected data with several mice reference atlases, such as NeuroTerrain [17], Minimum Deformation [18], and Waxholm [19] atlases. Once the region of interest has been identified, the researcher would then like to search for other image data that show expression patterns for gene lcn2, were collected to study Parkinson's disease, and contain the region of interest. As shown in Figure 3, the MBAT workflow to complete this use case would be as follows:

1. Using the Registration Workspace, the researcher aligns the collected image data to selected reference atlases to help identify the anatomic region of interest. The built-in registration methods include 2D to 2D or 2D to 3D alignment that use linear and non-linear algorithms.

2. Using the Comparison Viewer Workspace, the researcher can then overlay the aligned, collected image data with the reference atlases for comparison. The collected data can be compared to the reference atlases for differences around the unusual area. Also, the delineations for each individual atlas can be dynamically shown or hidden to help the researcher pinpoint the location of the unusual formation. Once candidate locations or anatomical structures, such as the putamen, have been identified, the researcher can initiate a search for these terms directly from the Comparison Viewer Workspace.

3. Using the Search Workspace, the researcher chooses which online data sources to search, such as gene expression images from ABA and GENSAT. The researcher can also refine the search criteria (putamen) by adding more terms, such as a gene name (lcn2) or the name of the disease (Parkinson's). Once the search results are returned, the researcher previews all the results from each of the data sources, and selects several for comparison.

4. Returning back to the Comparison Viewer Workspace, the researcher can automatically pull in the selected search results (gene expression images) to compare and contrast with the original collected data. This online image data can be juxtaposed or overlaid with the collected data and reference atlases. Alternatively, the online image data can be registered to the collected data or reference atlases prior to being loaded into the Comparison Viewer Workspace. As new hypotheses are sparked by comparing and associating data from multiple sources, this process of gathering, aligning, and correlating data can be repeated.

### Search Workspace Plug-ins

**Figure 4:**
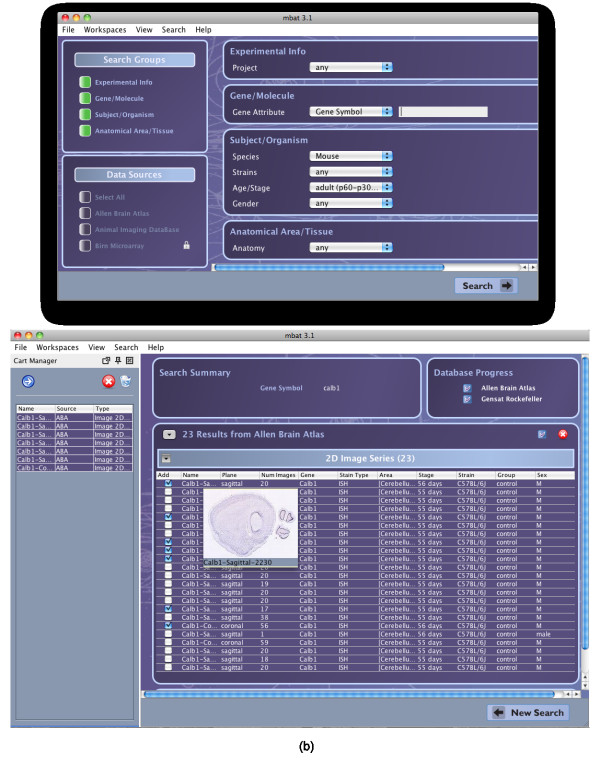
**MBAT Search Workspace Interface and Results**. The Search Workspace graphical user interfaces for the (a) query term subworkspace and (b) results page. The query term interface allows users to specify the query term parameters and choose which data sources to query. The results page groups the results by data source and data type with all the annotations in tabular form. Each result can be added to the global cart, as shown in the left side of (b). Hovering the mouse over the result will also show a thumbnail image to preview the result.

For the MBAT Search Workspace, two types of search plug-ins are defined, data and literature source plug-ins. The data source plug-ins access genomic and neuro-imaging data while the literature source plug-ins access publication records. As shown in Tables 2 and 3, the MouseBIRN programmers have, to date, developed 8 data source and 6 literature source plug-ins that access a variety of data - ranging from microarray probe data to gene expression and phenotype data to 2D and 3D image data. Many of the plug-ins connect to established and pre-existing data sources, such as the Allen Brain Atlas (ABA) and the Gene Expression Nervous System Atlas (GENSAT), without having to modify the original data source. Using the federated search engine, a user specifies the parameters for the single query and selects which data and literature sources will be searched. Examples of the query term interface and the results table are shown in Figure 4.

**Table 2:** MBAT Search Workspace Data Source Plug-ins.

Source	Method & Access	Search Types	Data Types
Allen Brain Atlas (ABA)	Web API (public)	Query Term	2D Image Series
Animal Imaging Database (AIDB)	Webservice (public)	Keyword, Query Term	3D Volume
BIRN Microarray Database	Webservice (public, private)	Query Term	Probe
Cell Centered Database (CCDB)	Webservice (public)	Keyword, Query Term	2D Image
CalTech Electronic Lab Notebook	Oracle JDBC (private)	Keyword	3D Volume
GENSAT Rockefeller	SOAP (public)	Query Term	2D Image Series, Gene, Gene Expression
GENSAT (NCBI Entrez)	Web (public) API	Keyword	2D Image
Gene Network	Webservice (public)	Keyword	Gene Expression, Phenotype

Summary of the Search Workspace data source plug-ins, listing the connection method to the underlying data source, the types of search methods supported, and the data types of the returned results.

**Table 3:** MBAT Search Workspace Literature Source Plug-ins.

Source	Access
Go3R	public
Google Scholar	public
GoPubMed	public
IEEE Explorer	private
PubMed	public
Springer Link	public

Summary of the Search Workspace literature plug-ins, listing the access type. All literature plug-ins use keyword search and the results are opened by the user's default web browser.

To allow for easy browsing and selection of the results, the search engine displays the returned values in tables, grouped by data source and data type, with each column displaying an annotation. Since the annotations for the same data type may vary across each data source, the data source plug-ins are allowed to return any number and type of annotations, such as plain text, numerical data, and URLs. To facilitate the browsing of the results, each annotation column can be dynamically shown or hidden, the results can be sorted by any annotation column, the annotation columns can be arbitrarily reordered, and the data source and data type groupings can be expanded or collapsed for easier viewing. For workspace interoperability, any of the query results can be placed into the cart so other workspaces have access to it. For example, a user can search for data in the Search Workspace, place the results into the cart, and then automatically download the data to analyze it in the Comparison Viewer Workspace.

### Registration Workspace plug-ins

**Figure 5:**
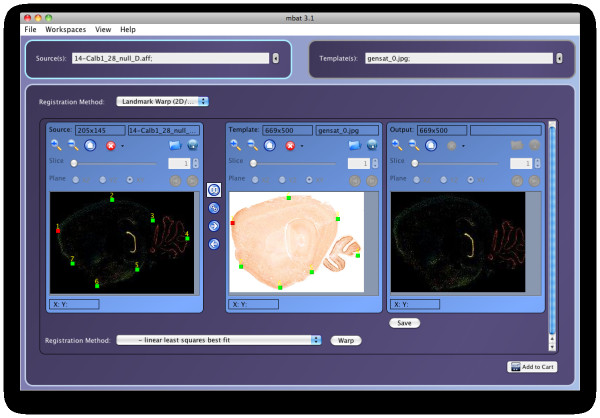
**MBAT Registration Workspace Interface**. The Registration Workspace showing the results of the LONI Landmark Warp Plug-in. A gene expression image is selected as the source image and shown on the left. The template image is shown in the center, with the paired landmarks overlaid on both the source and template images. The result of the landmark-based registration is shown on the right.

For the MBAT registration workspace, as shown in Table 4, there are currently two plug-ins - one that uses a local Java library and one that executes on a remote server. The Landmark Warp plug-in is a landmark based registration algorithm that uses the LONI Landmark Warp tools [20] to perform rigid body, affine, and non-linear spline based alignment. The AIR Pipeline plug-in is a workflow designed at the CalTech MRI Center at the Beckman Institute for aligning sets of mouse image volumes and contains three steps: (1) creating templates, (2) register pre-injection, and (3) register post-injection. It leverages the LONI Pipeline [21] and the Automated Image Registration (AIR) software [22] to batch and execute these workflows on a remote LONI Pipeline server. An example of the Registration Workspace and LONI Landmark Warp plug-in is shown in Figure 5.

**Table 4:** MBAT Registration Plug-ins.

Registration Plug-in	Type	Execution
Landmark Warp	Landmark based	Java (local)
AIR pipeline	AIR linear algorithms	LONI Pipeline (remote)

Summary of the Registration Workspace plug-ins, listing the type of algorithm used and the execution type.

### Comparison Viewer plug-ins

**Figure 6:**
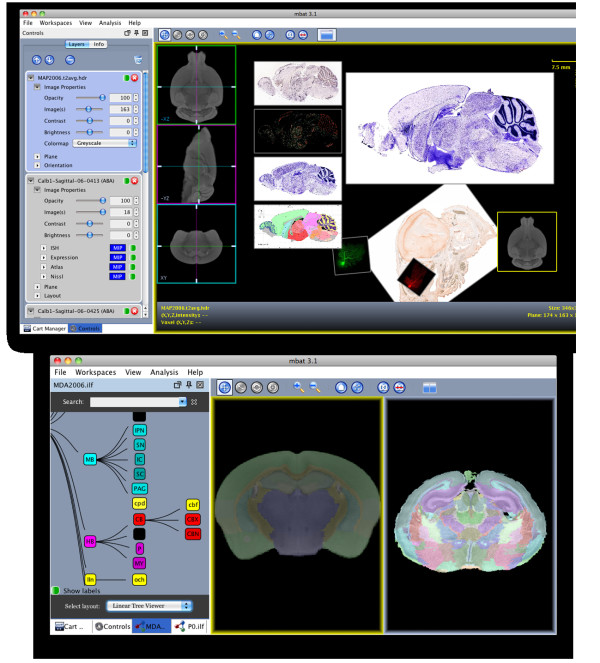
**MBAT Comparison Viewer Workspace Examples**. Examples of the layout, user interface, and plug-ins of the Comparison Viewer Workspace, showing (a) multiple layers composited together in a single canvas and (b) side-by-side comparison of a minimum deformation atlas (MDA) and pre-natal (P0) atlas using the dual-view mode and graph viewer. In the top image, clock-wise from the left, the plug-ins used are (1) the Allen Brain Atlas (ABA) plug-in displaying in situ, gene expression, Nissl, and the ABA atlas as one synchronized layer, (2) the 2D image plug-in displaying a sagittal series of ABA Nissl images, (3) the 3D volume plug-in displaying a MR volume of a mouse brain with the orthogonal plane controls displayed at the left, and (4) the 2D image plug-in displaying single images directly downloaded from the GENSAT and CCDB databases.

As shown in Table 5, a mix of plug-ins for a variety of data types and uses were developed for the Comparison Viewer Workspace. The core layer rendering plug-ins for basic data types are shown in Figure 6a and include the plug-ins for 2D images, 3D image volumes, and 3D atlases. The 2D Image plug-in reads common 2D image formats and uses the dlGraphics mid-level library to manage and render individual or series of 2D images. The 3D Volume plug-in officially reads Analyze and NIfTI file formats and extends the 2D Image plug-in by streaming the orthogonal slice data from memory as virtual image series. Orthogonal views of the major axes of the 3D volume can be interactively navigated using our heads-up-display controls. The 3D Atlas plug-in extends the 3D Volume plug-in to add overlaying and streaming of the label volume from memory. The 3D Atlas plug-in is described in more detail below.

**Table 5:** MBAT Comparison Viewer layer rendering plug-ins.

Layer Rendering plug-in	File types	Cart objects	Add Layer
2D Image	2D files (individual or as series)	Image2D, Image2DSeries, BufferedImageObject	Yes
3D Volume	3D files	Volume3D	No
3D Atlas	.atlas files	None	No
NeuroTerrain (remote image server)	None	None	Yes
Mouse Brain Library (remote image server)	None	Nonex	Yes
Large Volume	Large Analyze Image (.lhdr)	None	No
Large Volume Atlas	.atlas (using .lhdr)	None	No
ABA	None	ABA images	No
GENSAT	None	GENSAT images	No

Summary of the layer rendering plug-ins for the Comparison Viewer Workspace, listing the file types and cart objects that can be opened and if adding a layer is supported.

The Large Volume, NeuroTerrain, and Mouse Brain Library (MBL) plug-ins all extend the 3D Volume plug-in to load custom data provided by the MouseBIRN collaborators. The Large Volume plug-in handles 3D image data that is too large to fit into main memory by using an on-demand streaming technique that reads the image data directly from files on disk. The Large Volume Atlas plug-in uses the same out-of-core technique to extend the 3D Atlas plug-in for atlases that cannot fit into main memory. The NeuroTerrain and MBL plug-ins leverage the NeuroTerrain Client-Server Atlas System [23] to add streaming of image data through a remote image server.

As shown in Table 6, each of these rendering plug-ins can be overlaid and juxtaposed in the virtual canvas. Every plug-in layer can be panned, rotated, scaled, and zoomed. A user can also adjust each layer's opacity, contrast, and brightness through the user interface widgets that control the properties of the layer, as shown in the left of Figure 6a. Linking layers is another key feature where rendering layers are synchronized together, with slave layers mirroring the actions applied to the master layer. Linking layers makes it easier to match views for comparison. Another tool to help user's compare data is the dual-view mode which helps organize the layers for easier juxtaposition of data, as shown in Figure 6b. Each rendering plug-in can also define custom actions for interrogating the layer. For example, for the 3D Volume and Atlas plug-ins, right-clicking on the image allows the user to automatically send the current image location or underlying label to the Search Workspace for querying.

**Table 6:** MBAT Layer Rendering Plug-ins Feature List.

Feature	2D	2D series	3D	3D Atlas
Pan	✔	✔	✔	✔
Zoom	✔	✔	✔	✔
Rotate	✔	✔	✔	✔
Scale	✔	✔	✔	✔
Contrast	✔	✔	✔	✔
Brightness	✔	✔	✔	✔
Overlay	✔	✔	✔	✔
Opacity	✔	✔	✔	✔
Link with layers	✔	✔	✔	✔
Image coordinates under mouse	✔	✔	✔	✔
Cine images		✔	✔	✔
Orthogonal views			✔	✔
Reorient volume			✔	✔
Change color lookup table			✔	✔
Coordinate system transformation			✔	✔
Origin markers			✔	✔
Send location to search			✔	✔
Send label to search				✔
Label and group selection				✔
Set background color				✔
Mask/unmask background				✔
Multiple label sets				✔
Show active label under mouse				✔
Selected labels highlighted in graph viewer				✔
Selected labels in graph viewer highlighted				✔

Summary of the features for the core layer rendering plug-ins of 2D images, 2D image series, 3D volumes, and 3D atlases.

As shown in Table 7, two graph viewer plug-ins were developed that support the ILF format. The ILF format currently supports hierarchical relationships with single parents. The graph viewer plug-ins are synchronized with the 3D Atlas plug-in to highlight the selected labels. Both graph viewer plug-ins also provide search features that automatically highlight labels based on matching text patterns in either the label abbreviation or full name.

**Table 7:** MBAT Graph Viewer plug-ins.

Name	Format	Layout	Search
Radial Graph	ILF	Each generation placed on concentric circles	Yes
Linear Graph	ILF	Each generation placed to the right of the previous generation	Yes

Summary of the graph viewer plug-ins used in the Comparison Viewer Workspace, listing the format supported, layout of the graph, and if the nodes are searchable.

#### Atlas Plug-in

**Figure 7:**
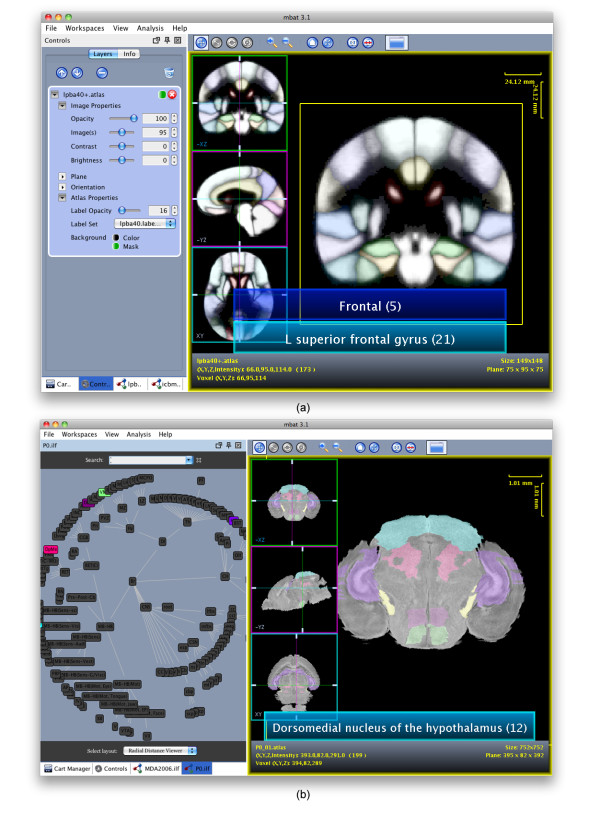
**3D Atlas plug-in**. Examples of the interactive features of the 3D Atlas plug-in, showing (a) the multiple label display for a human probabilistic atlas and (b) the synchronization between the 3D Atlas plug-in in the Comparison Viewer and the graph viewer for P0 atlas, with the selected structures highlighted in both displays.

The 3D Atlas plug-in requires a label 3D volume that labels each voxel with a label ID and a label hierarchy file that defines the label ID to text description and color lookup table. Using the label location and meta-data, the 3D Atlas plug-in can overlay the colored regions with user controlled transparency over a regular reference 3D volume, such as MR data, and display the label description, as shown in Figures 6b and 7.

Unique to our atlas plug-in is the ability to have multiple label files for a given atlas. This allows the user to define different labels for the same location for different contexts. For example, a location may have a structure name in an anatomic context but a biological purpose in a functional context. Hovering the mouse pointer over a valid label region displays the labels for that given location. If multiple labels are present, all labels for that location are displayed, with the active label set displayed first, as shown in Figure 7b. Other interactive features include dynamic selection and display of the segmented atlas label regions. Individual label or groups of regions can be enabled and disabled by simply double-clicking the area with the mouse.

Another novel informatics feature of the 3D Atlas plug-in is the synchronization between the visual atlas and the ontological relationships displayed in the graph viewer. Whenever a label region is selected in the rendering layer of the 3D Atlas plug-in, the corresponding node in the ontology is highlighted by the graph viewer plug-in. For example, in Figure 7b, the Dorsomedial nucleus of the hypothalamus is one of the structures selected in the 3D Atlas plug-in so consequently the DMH node in the graph viewer is highlighted. The synchronization is bi-directional, so selecting any nodes in the graph viewer will also highlight them in the 3D Atlas rendering plug-in. For example, selecting a parent node, such as the forebrain, will automatically select all the sub-structures in the ontology graph, such as the amygdala, basal ganglia, and cerebral cortex, and highlight them in the Comparison Viewer. In this way, the researcher can easily use a physical location to lookup labels and vice versa, use ontological relationships to locate the label position in the atlas during the discovery process.

### Plug-in Framework

The MBAT tiered plug-in architecture uses the Java Plugin Framework (JPF) [24] to manage the plug-ins. JPF is an open source library designed to provide a standard plug-in infrastructure for Java projects. It includes a runtime engine that dynamically discovers, instantiates, and runs plug-ins. JPF also supports hot deployable plug-ins that can be started and stopped at runtime without restarting the entire system. The design and implementation of JPF are inspired and influenced, but not derived, from the original, proprietary Eclipse plug-in framework. JPF decouples the plug-in infrastructure from the main Eclipse infrastructure into a standalone library. JPF was chosen since it is extremely lightweight, has a low learning curve, and is extremely flexible and extensible.

The Eclipse plug-in framework has since adopted the OSGi specification [25], a standard developed by a world wide consortium of technology vendors to facilitate the modularization of Java software components and assure the interoperability among applications. While the syntax and configuration of JPF and OSGi are incompatible, the semantics are very similar. Hence, for plug-ins designed modularly, the conversion from JPF to OSGi is straight-forward and does not require major code rewriting. Future versions of MBAT will benefit from switching plug-in frameworks as it will make it immediately interoperable with OSGi applications, such as the chemo- and bioinformatics plug-ins found in Bioclipse [26,27].

## Conclusions

We have presented a tiered, plug-in architecture that has been tailored to a neuroinformatics and genomics application, MBAT. MBAT employs the Search, Registration, and Comparison Viewer workspaces to accelerate the tasks that compromise the digital atlas analysis workflow - gathering, alignment, and visualization of disparate data. We have shown how data widely available through distributed online resources can be integrated using the federated search, registration, compositing, and atlasing tools of MBAT. Through the plug-ins, we have shown how functionality can be extended and how parts of existing applications can be leveraged and integrated into MBAT to create personalized workflows. The broad range of plug-in types also shows the versatility and flexibility of the tiered, plug-in architecture.

Through the 3D Atlas plug-in, we have introduced novel digital atlas analysis tools that allow multiple categorizations of label regions, dynamic selection and grouping of labels, and context-specific display of informatics data through the synchronization of the rendering layer and graph viewer plug-ins. Future work includes extending the Comparison Viewer Workspace to include data that has been semantically registered to an atlas, extending the graph viewer plug-ins to support more ontological relationship types, extending the search engine to allow selection of query terms that are specific to a particular data source, and extending the Registration Workspace to allow real-time comparison of the results of different algorithms. With the continued collaborative development of MBAT workspaces and tool plug-ins, we believe this can lead to a self-sustaining and rich library of digital atlasing tools.

## Availability and requirements

• Project Name: MBAT

• Project home page: http://mbat.loni.ucla.edu

• Operating System(s): Platform independent

• Programming language: Java

• Other requirements: Java 1.6+, OpenGL 1.5+

• License: BIRN License

## List of abbreviations used

API: Application Programming Interface; BIRN: Biomedical Informatics Research Network; MBAT: MouseBIRN Atlasing Toolkit.

## Authors' contributions

DL is the architect of the core framework, designed the workspaces, and wrote this manuscript. DL, QN, NS, SA designed and implemented the workspace cores and the majority of the plug-ins. SR, the Program Manager of the MouseBIRN testbed, and AT, the Principle Investigator, provided the vision for this project. All authors read and approved the final manuscript.
